# Quantitative Proteomic Analysis of Tibetan Pig Livers at Different Altitudes

**DOI:** 10.3390/molecules28041694

**Published:** 2023-02-10

**Authors:** Xuedong Gu, Xinping Chang, Lin Yang, Yangzom Chamba, Fang Geng

**Affiliations:** 1 Institute of Tibet Plateau Ecology, Tibet Agriculture and Animal Husbandry University, Linzhi 860000, China; 2 The Provincial and Ministerial Co-Founded Collaborative Innovation Center for R & D in Tibet Characteristic Agricultural and Animal Husbandry Resources, Linzhi 860000, China; 3 Meat Processing Key Laboratory of Sichuan Province, School of Food and Biological Engineering, Chengdu University, Chengdu 610106, China

**Keywords:** Tibetan pig, proteomics, liver, lipid metabolism, immunoglobulin

## Abstract

In this study, the differences in protein profiles between the livers of Shannan Tibetan pigs (SNT), Linzhi Tibetan pigs (LZT) and Jiuzhaigou Tibetan pigs (JZT) were comparatively analyzed by tandem mass spectrometry-labeling quantitative proteomics. A total of 6804 proteins were identified: 6471 were quantified and 1095 were screened as differentially expressed proteins (DEPs). Bioinformatics analysis results show that, compared with JZT livers, up-regulated DEPs in SNT and LZT livers mainly promoted hepatic detoxification through steroid hormone biosynthesis and participated in lipid metabolism to maintain body energy homeostasis, immune response and immune regulation, while down-regulated DEPs were mainly involved in lipid metabolism and immune regulation. Three proteases closely related to hepatic fatty acid oxidation were down-regulated in enzymatic activity, indicating higher levels of lipid oxidation in SNT and LZT livers than in JZT livers. Down-regulation of the expression of ten immunoglobulins suggests that JZT are more susceptible to autoimmune diseases. It is highly likely that these differences in lipid metabolism and immune-related proteins are in response to the ecological environment at different altitudes, and the findings contribute to the understanding of the potential molecular link between Tibetan pig livers and the environment.

## 1. Introduction

The Tibetan pig, a plateau miniature pig breed, mainly comes from the Qinghai–Tibetan plateau in China [1,2]. Due to natural and artificial selection under a long-term low-oxygen and high-altitude environment, Tibetan pigs have genetic characteristics and living habits that are different from those of ordinary domestic pigs [3,4]. Prolonged exposure to a high-altitude hypoxic environment has caused the livers of Tibetan pigs to gradually develop a stronger adaptation capacity against stress, thus showing a stronger resistance to disease [5]. In addition, the chemical element composition of the Tibetan pig liver is closely related to its growing environment and feed consumption [6]. Therefore, the liver metabolic activity of Tibetan pigs can further reflect the ecology of the plateau.

Tibetan pigs raised in natural pastures not only have unique meat attributes and eating quality, but are also rich in a variety of nutrients [7,8]. In recent years, with the rapid development of the Tibetan pig breeding industry, the topography of its breeding area is complex and diverse, which can be roughly divided into high mountainous areas (above 3000 m), semi-mountainous areas (2000–3000 m) and river valley areas (below 2000 m). In this sense, the feeding patterns of Tibetan pigs also differ, leading to differences in liver metabolism in Tibetan pigs from various altitude breeding areas. In an earlier study, it was mentioned that the adaptations of an organism can influence the ecology and evolution of the species [9]. To some extent, this species identity also reflects ecological changes. Reportedly, Wang Yandong et al. [10] identified a gene involved in anti-hypoxia (ECE1) expressed in the liver tissue of Tibetan pigs with elucidated functions of this gene in their plateau adaptation ability. Additionally, Beji et al. [11] determined that when tissue oxygen supply is insufficient, the increased expression of the erythropoietin (EPO) gene is a molecular mechanism for the liver to adapt to the hypoxic environment. These studies explained the adaptability of the Tibetan pig liver to high-altitude hypoxic environments at the gene level.

In addition, the regulatory mechanism of microRNA in the liver metabolism of Tibetan pigs was discussed in a deep-sequencing comparison of the liver microRNA transcriptomes of Tibetan pigs and Yorkshire pigs [12]. It is well known that RNA is more stable when translated into proteins and performs the function of life activities in the organism. Therefore, a comprehensive histological analysis of the livers of Tibetan and Yorkshire pigs was performed by RNA sequencing and isotope-labeled relative and absolute quantification (iTRAQ), which characterized the metabolic adaptation of the liver to hypoxic conditions [13]. Additionally, researchers have identified 1723 proteins from the dorsal longissimus muscle of Tibetan pigs [14]. A quantitative proteomic comparison between Tibetan and Yorkshire pigs was also subsequently performed to dissect the meat quality differences between the two pork species [15]. However, although these studies can provide some theoretical basis for the adaptation of Tibetan pigs to hypoxia, they do not provide comprehensive information on the ecological development of the plateau because of genetic differences between Tibetan and Yorkshire pigs [16,17]. Therefore, it is important to explore protein characterization in the liver of Tibetan pigs from different altitude regions through proteomics to discover the potential connection between Tibetan pigs and the plateau ecology for the development of the plateau livestock industry in China.

The aim of this study was to perform quantitative proteomic analysis of liver tissues from Shannan Tibetan pigs (SNT, about 4000 m), Linzhi Tibetan pigs (LZT, about 3000 m) and Jiuzhaigou Tibetan pigs (JZT, about 1500 m). Therefore, a quantitative proteomics analysis workflow combining TMT labeling, high-pH reversed-phase high-performance liquid chromatography (RP-HPLC) and liquid chromatography–tandem mass spectrometry (LC-MS/MS) was used to compare the differences in protein profiles between three Tibetan pig livers. This study will reveal the molecular mechanisms underlying the differences in liver metabolism in Tibetan pigs under different altitudinal environments, which will help provide insights into the conservation of highland animals and ecological protection.

## 2. Results

### 2.1. Proteome of Tibetan Pig Liver

**Figure 1:**
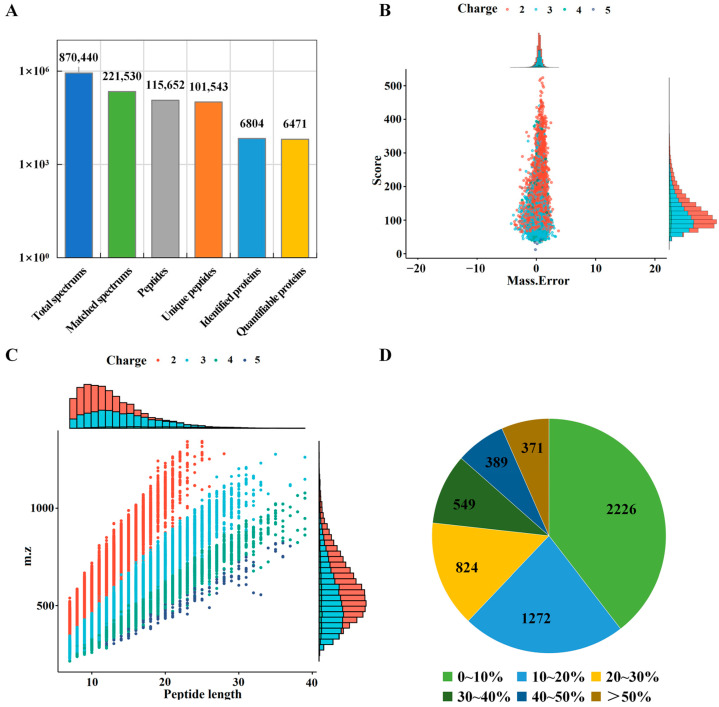
Characterization of identified proteins in Tibetan pig liver. (**A**) Statistical information of tandem mass spectra and protein identification; (**B**) number of identified peptides with mass error; (**C**) peptide length distribution; (**D**) protein coverage distribution map.

Quantitative proteomic analysis was used to explore the protein difference profiles among Tibetan pig livers at three altitudes. A total of 115,652 protein peptides were identified by LC-MS/MS from the livers of Tibetan pigs (Figure 1A). The vast majority of protein peptides had high precision characteristics with peptide ion mass error < 5 ppm (Figure 1B). Most of the peptides were distributed over 7–20 amino acids, in accordance with the general pattern based on trypsin digestion and HCD fragmentation mode (Figure 1C). The quality of protein identification was high, with more than half (60.5%) of the identified proteins having a sequence coverage higher than 10% (Figure 1D). In addition, 6804 proteins were identified, of which 6471 were quantifiable (Table S1), indicating the high quantification efficiency of the TMT marker. After collation and analysis, the TMT labeling efficiency values reached more than 96% for all three sets of replicate samples (Table S2). Finally, the reproducibility of TMT marker quantification was found to be excellent by relative standard deviation (RSD) and Pearson’s Correlation Coefficient analysis (Table S3, Figure S1).

### 2.2. Differentially Expressed Proteins (DEPs)

**Figure 2:**
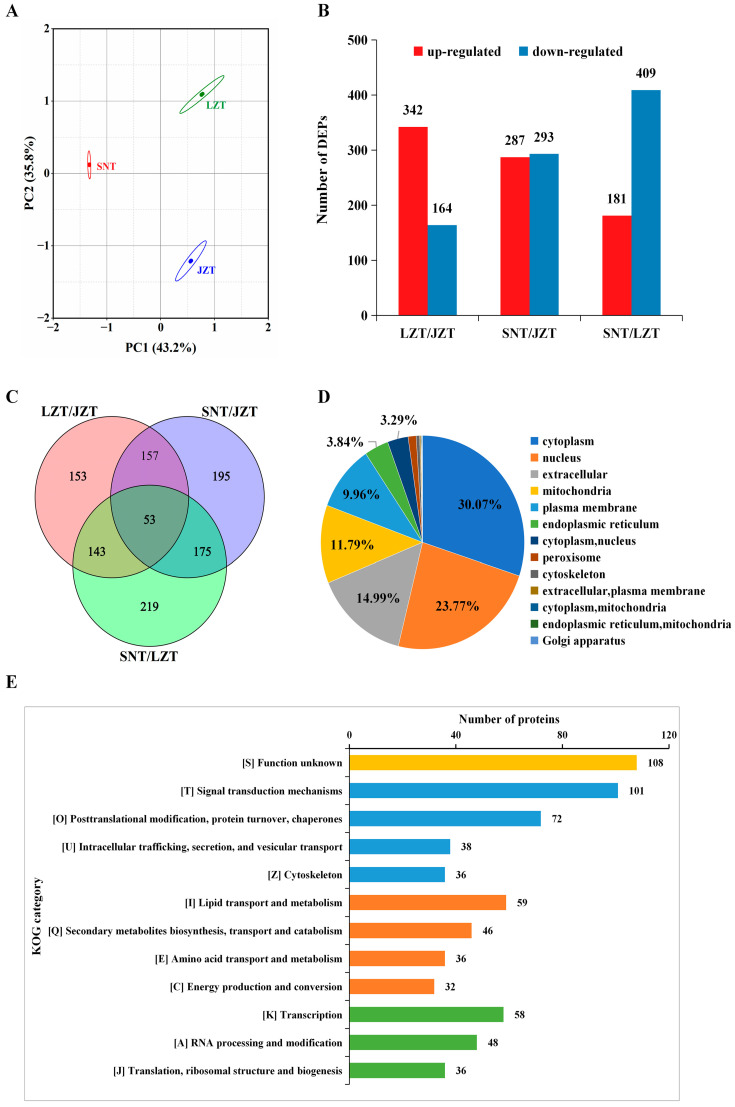
Characteristics and annotation of differentially expressed proteins (DEPs) in Tibetan pig liver. (**A**) Principal component analysis (PCA) analysis of the proteomic profiles of three Tibetan pigs; (**B**) the distribution of DEPs in three groups of Tibetan pig livers; (**C**) Venn diagram of DEPs in three component-pair comparisons; (**D**) subcellular localization annotation of DEPs; (**E**) COG/KOG annotation of DEPs.

Multivariate analysis of the proteomic data was performed to further understand the differences between Tibetan pig livers at the three altitudes. The results of principal component analysis (PCA) identified differences between the SNT, LZT and JZT liver proteomes, with principal component 1 (PC1) and principal component 2 (PC2) values of 43.2% and 35.8% for the three Tibetan pig liver samples, respectively (Figure 2A). A total of 1095 differentially expressed proteins (DEPs) were screened according to *p* ≤ 0.05 and change ratio of differential expression of protein > 1.3 (or <0.77), with details shown in Table S4. Additionally, pairwise comparisons between three groups of Tibetan pig livers were performed, and the results showed that 342 were up-regulated and 164 down-regulated in LZT/JZT; 293 up-regulated and 283 down-regulated in SNT/JZT; and 181 up-regulated and 409 down-regulated in SNT/LZT (Figure 2B). This indicated that protein expression differed significantly between the three Tibetan pig livers. Furthermore, the Venn of DEPs in LZT/JZT, SNT/JZT and SNT/LZT showed that only 53 DEPs (4.8%) were present in all three comparisons simultaneously, while the highest number of DEPs was specific to SNT/LZT, followed by SNT/JZT (Figure 2C). These results further suggest that protein expression differences between the three Tibetan pig livers are large.

### 2.3. Annotation Analysis of DEPs

#### 2.3.1. Subcellular Localization

The subcellular localization of DEPs in the livers of three Tibetan pigs was predicted to obtain more information. A total of 1094 proteins were annotated in subcellular localization, and these proteins were mainly localized in the cytoplasm (30.07%) and nucleus (23.77%). In addition, 14.99% of the proteins were distributed outside the cell, and 11.79% and 9.96% were localized to the mitochondria and plasma membrane, respectively (Figure 2D). To understand the subcellular localization of DEPs among the three groups of Tibetan pig liver contrasts, they were predicted. The results showed that the up-regulated DEPs in LZT/JZT were mainly distributed in the cytoplasm and nucleus, while the down-regulated DEPs were in the cytoplasm and extracellular regions; both the up-regulated and down-regulated DEPs in SNT/JZT were concentrated in the cytoplasm and extracellular regions; the cytoplasm and extracellular regions were the main localization regions of the up-regulated DEPs in SNT/LZT, while the down-regulated DEPs were mainly distributed in the nucleus and cytoplasm (Figure S2A). In summary, protein localization in SNT and JZT livers is similar, mainly in the cytoplasm and extracellular, while proteins in LZT livers are mainly distributed in the cytoplasm and nucleus, which may lead to different physiological functions in LZT livers than in SNT and JZT livers.

#### 2.3.2. COG/KOG Annotations

The KOG categories annotated to the top 12 DEPs were screened from three Tibetan pig livers, and the KOG subclasses with the highest number of DEPs were found to be [S], [T], [0], [I] and [K] (Figure 2E). These KOG subclasses mainly include signal transduction mechanisms, posttranslational modification, protein turnover, chaperones, and lipid transport, metabolism and transcription. Among them, the largest number of proteins (108) were annotated in “[S] Function unknown”, suggesting that these proteins may be unique energy-supplying proteins in Tibetan pig liver metabolism and deserve to be explored in depth. Consistently, the same results appear in the three groups compared in pairs (Figure S2B). “[T] Signal transduction mechanisms” was the main KOG item for DEPs up-regulated in LZT/JZT, while down-regulated DEPs were mainly associated with “[I] Lipid metabolism and transport”. “[I] Lipid metabolism and transport” was the main KOG item for DEPs up-regulated in SNT/JZT, while down-regulated DEPs were mainly associated with “[T] Signaling mechanisms”; the annotated KOG results of up-regulated and down-regulated DEPs in SNT/LZT were consistent with SNT/JZT. In conclusion, proteins in SNT livers are mainly highly expressed in “Lipid metabolism and transport”, while proteins in LZT liver are involved in “Signaling mechanisms”.

#### 2.3.3. GO Annotations of DEPs

**Figure 3:**
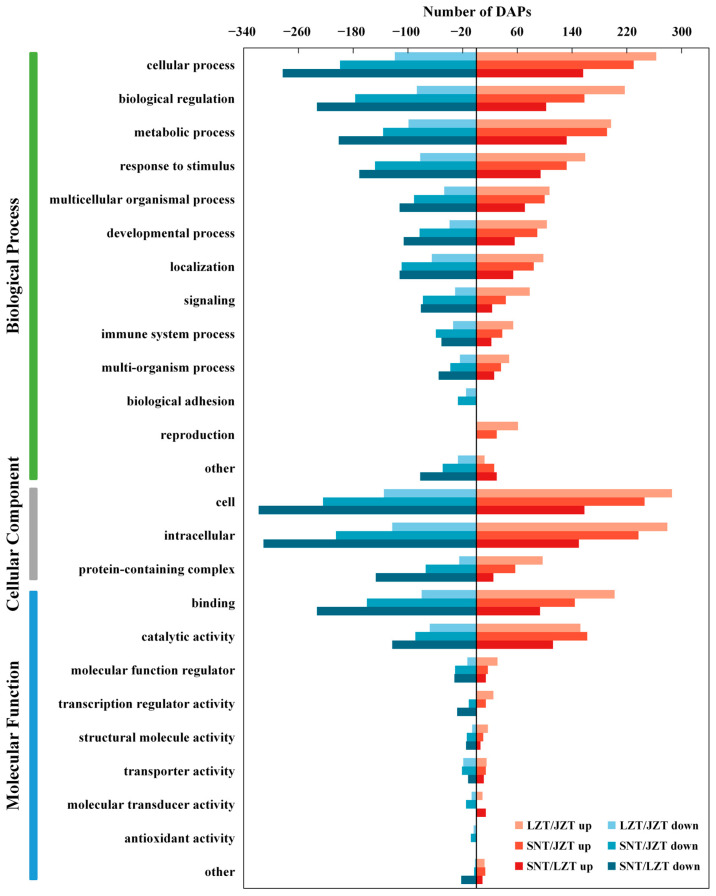
GO annotation of DEPs in LZT/JZT, SNT/JZT and SNT/LZT.

In this study, the GO terminology annotation allowed the classification of DEPs in the three Tibetan pig livers into three categories: biological processes (BP), cellular components (CC) and molecular functions (MF) (Figure 3). First, the DEPs annotated in the “BP” category were mainly related to “cellular process”, “biological regulation”, “metabolic process” and “response to stimulus”. It is noteworthy that, among these four GO terms, there were more up-regulated DEPs than down-regulated DEPs in LZT/JZT, while the opposite result was annotated for DEPs in SNT/LZT; that is, there were fewer up-regulated DEPs than down-regulated DEPs. Second, for the “CC” category, DEPs were mainly distributed in the “cell” and “intracellular” categories. Specifically, up-regulation of DEPs annotated as “cellular” and “intracellular” occurred in SNT/JZT and LZT/JZT, while DEPs were more down-regulated in SNT/LZT. In addition, “binding” and “catalytic activity” were the most representative GO terms for the three Tibetan porcine livers in the “MF” category. Interestingly, DEPs in SNT/JZT were more down-regulated than up-regulated in the “binding” term, and more up-regulated DEPs than down-regulated DEPs in the “catalytic activity” term.

### 2.4. Enrichment Analysis of DEPs

#### 2.4.1. GO Enrichment Analysis

To further assess the physiological activities involved in the DEPs in the livers of the three Tibetan pigs, and thus their response to the ecological environment, GO enrichment analysis was performed (Table S5). To begin with, the up-regulated DEPs in the SNT/JZT and SNT/LZT were mainly involved in two types of “BP”, namely, carboxylic acid metabolism and lipid metabolism. The GO terms related to carboxylic acid metabolism included “carboxylic acid metabolic process” (GO:0019752), “monocarboxylic acid metabolic process” (GO:0032787) and “xenobiotic metabolic process” (GO:0006805), while GO related to lipid metabolism included “regulation of lipid metabolic process” (GO:0019216), “steroid biosynthetic process” (GO:0006694), “secondary alcohol metabolic process” (GO:1902652) and “sterol metabolic process” (GO:0016125). In the “MF” category, 68 and 47 up-regulated DEPs in the SNT/JZT and SNT/LZT, respectively, had “oxidoreductase activity”, and 31 and 23 up-regulated DEPs, respectively, were enriched in the GO term of “cofactor binding”. Most of the up-regulated DEPs were involved in “CC” in the “endoplasmic reticulum”, while a few of them were active in the “peroxisome”. In addition, 56 up-regulated DEPs in the LZT/JZT were involved in the “BP” of “carboxylic acid metabolism” (GO:0019752), while the remaining DEPs were mainly involved in xenobiotic metabolic processes and sterol biological metabolism, such as “xenobiotic metabolic process”, “cellular hormone metabolic process”, “secondary alcohol metabolic process” and “sterol metabolic process”, etc. Up-regulated DEPs of LZT had multiple “MF”, namely “monooxygenase activity” (GO:0004497), “steroid hydroxylase activity” (GO:0008395), “DNA-dependent ATPase activity” (GO:0008094) and “NADH binding” (GO:0070402). In the “CC” category, LZT mainly dealt with GO terms related to nucleic acid repair.

Surprisingly, the involvement of down-regulated DEPs in the LZT/JZT and SNT/JZT was similar in terms of physiological activities. For example, the “BP” category, involved in lipid metabolism, mainly included “regulation of lipid metabolic process” (GO:0019216 and GO:0019217), “lipid transport” (GO: 0006869), “protein-containing complex remodeling” (GO:0034367) and 14 other GO terms. Differently, down-regulated DEPs in SNT/JZT were enriched for immune-related GO terms, including “protein activation cascade” (GO:0072376) and “blood coagulation, fibrin clot formation” (GO:0072378). The main “CC” terms of DEPs down-regulated in LZT/JZT and SNT/JZT were “extracellular region” (GO:0005576 and GO:0005615) and “blood microparticle/chylomicron” (GO:0072562/GO:0042627). In the “MF” category, the down-regulated DEPs were enriched for GO items such as “lipid transporter activity” (GO:0005319), “cholesterol binding” (GO:0015485) and “sterol binding” (GO:0032934). Notably, 17 down-regulated DEPs in the LZT/JZT were enriched for the “cofactor binding (GO:0048037)” GO term. In addition, for the 409 down-regulated DEPs in the SNT/LZT, 67 DEPs were involved in the “RNA metabolic process” (GO:0016070) and 78 DEPs were involved in the “nucleic acid metabolic process” (GO:0090304) in the “BP” category. Most of the down-regulated DEPs were enriched in the “CC” category for GO terms including “supramolecular fibers” (29 DEPs), “focal adhesion” (14 DEPs) and “cell-substrate junction” (14 DEPs), etc. In addition, the down-regulated DEPs in SNT/LZT were closely related to the GO terms “nucleic acid binding (GO:0003676)”, “RNA binding” (GO:0003723) and “DNA binding (GO:0003723)” in the “MF” category.

#### 2.4.2. KEGG Enrichment Analysis

**Figure 4:**
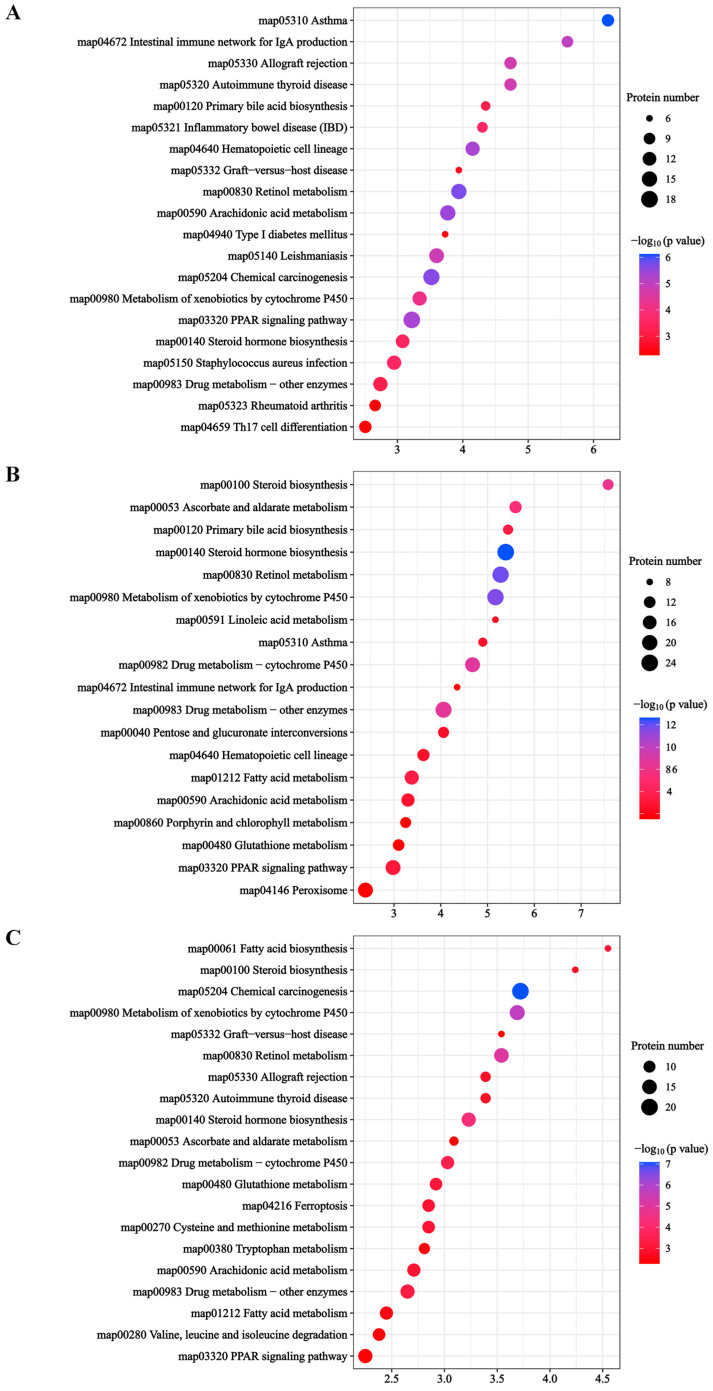
KEGG enrichment results of DEPs in LZT/JZT (**A**), SNT/JZT (**B**) and SNT/LZT (**C**).

The KEGG pathway of DEP enrichment in the livers of three Tibetan pigs was analyzed to further understand the differences in liver physiological metabolism in Tibetan pigs under different plateau environments. The results of KEGG enrichment showed that a total of 642 DEPs were enriched in the KEGG pathway. There were 307 DEPs enriched in 43 KEGG pathways in the LZT/JZT; 351 DEPs were also enriched in 43 KEGG pathways in the SNT/JZT, while 342 DEPs were enriched in 41 KEGG pathways in the SNT/LZT (Table S6). The top 20 KEGG pathways with the most significant enrichment of DEPs in the LZT/JZT were screened (Figure 4A). The enrichment results showed that most DEPs were involved in disease-related metabolic pathways; for example, “asthma” (map05310), “leishmaniasis” (map05140), “autoimmune thyroid disease” (map05320), “inflammatory bowel disease” (map05321) and “Staphylococcus aureus infection” (map05150), among 12 other pathways. Interestingly, the core proteins in these pathways were found to be immune-related after analysis of these DEPs, and enriched in the GO term of “extracellular domain”. In addition, DEPs from this group were also involved in detoxification metabolism (six pathways, including “retinol metabolism”, “chemical carcinogenesis”, and “cytochrome P450 metabolism of xenobiotics”) and lipid metabolism. In particular, most of the DEPs involved in detoxification metabolism were up-regulated, while the DEPs involved in lipid metabolism were down-regulated, which was consistent with the GO enrichment results of DEPs in LZT/JZT.

In addition, the first 20 KEGG pathways enriched for DEPs in the SNT/JZT and SNT/LZT groups were similar (Figure 4B,C). These DEPs were mainly involved in detoxification metabolism, lipid metabolism and immune defense. Detoxification metabolism mainly included “chemical carcinogenesis” (map05204), “retinol metabolism” (map00830), “steroid hormone biosynthesis” (map00140), “cytochrome P450 metabolism of xenobiotics” (map00980) and “drug metabolism-cytochrome P450” (map00982); closely related to the lipid metabolism are “fatty acid metabolism” (map01212) and “PPAR signaling pathway” (map03320). Unlike the immune-related KEGG pathway, the enriched DEPs were all from the SNT/JZT group, such as “hematopoietic cell lineage” (map04640) and “asthma” (map05310). These metabolic pathways are consistent with the GO enrichment results, indicating that the physiological activities of Tibetan pig livers are closely related to different altitude ecological environments, which will be further discussed in detail.

## 3. Discussion

### 3.1. Steroid Hormones Promote Liver Detoxification

Exposure to alpine hypoxia affects the metabolic levels of steroid hormones in the livers of Tibetan pigs. Steroid hormones are a class of lipid-soluble signaling molecules synthesized and released by the hypothalamic–pituitary–adrenal/gonadal axis, which can bind to nuclear receptor proteins through the plasma membrane of target cells and alter the transcription of specific genes, thereby regulating important physiological functions in the organism. A previous study reported that steroid hormones play an important role in the regulation of water and salt homeostasis, metabolism and oxidative stress, and immunity, growth and development [18]. Based on quantitative proteomics, the regulation of steroid hormones may be crucial to the physiological homeostasis of Tibetan pigs in a plateau environment. This study suggests that the regulation of steroid hormones may be critical to the physiological homeostasis of Tibetan pigs in response to the plateau environment. Meanwhile, the KEGG pathway of “steroid hormone biosynthesis” was found to be significantly enriched in the livers of three Tibetan pigs (Figure 4). All steroid hormones are synthesized from cholesterol as the starting material through the action of two key enzymes: cytochrome oxidases P450 (CYPs) and steroid hydroxy dehydrogenases (HSDs). Specifically, cholesterol side chains are catalyzed by CYP11A cleavage to produce pregnenolone, which is then metabolized to other steroids via HSD conversion; in particular, HSD3B, which—together with CYP17A1, a key branch point in steroid biosynthesis—directs the production of corticosteroids or sex hormones [19]. In addition, the catalytic metabolism of CYP21A produces glucocorticoids and salt corticoids. The former mainly affect glucose metabolism and promoting gluconeogenesis; the latter are mainly involved in salt metabolism and regulating blood electrolyte concentration.

**Figure 5:**
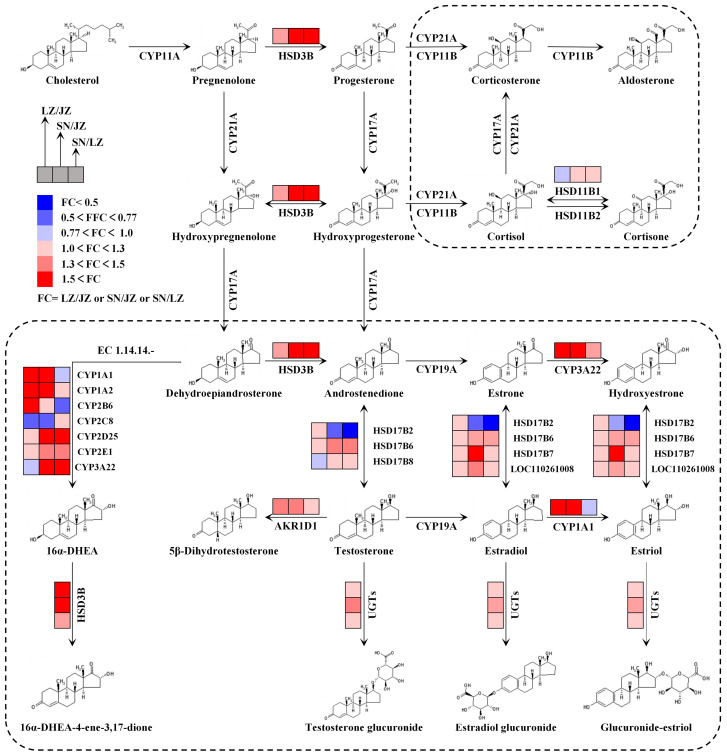
The protein expression in the liver of Tibetan pigs in the “steroid hormone biosynthesis” pathway.

Notably, the sex hormone metabolic branch of the three Tibetan pig livers differed significantly in the “steroid hormone biosynthesis” pathway (Figure 5). In detail, three important classes of metabolic enzymes appeared to be differentially expressed in the livers of three Tibetan pigs, including HSDs, CYPs and glucuronosyl transferase (UGT). First, HAD17Bs are a group of enzymes that catalyze the interconversion between 17-steroids and 17-sitosterones. Among them, HSD17B2 is the key HSD17B isoenzyme that inactivates estrogens and androgens, and it is expressed down-regulated in SNT livers and works together with the highly expressed HSD17B6, HSD17B7 and HSD17B8 to participate in sex hormone bioregulation, and in the metabolism of cholesterol biosynthesis, bile acids and fatty acids [20,21]. Steroid 5β-reductase (AKR1D1) also belongs to the HSD family of enzymes, and knockdown of the AKR1D1 gene decreases bile acid biosynthesis and steroid hormone clearance [22]. Therefore, the high expression of AKR1D1 in the livers of SNT and LZT will promote the metabolism of endogenous agents. Secondly, CYPs and UGTs are xenobiotic metabolic enzymes capable of reducing the accumulation of toxic endogenous chemicals via constitutive androstane receptors [23]. CYPs, the most critical enzyme in phase I metabolism of biotransformation, was not only enriched in the KEGG pathway of “steroid hormone biosynthesis”, but also significantly enriched in GO terms such as “metabolism of xenobiotics”, “cellular hormone metabolic process” and “catabolic process of xenobiotics”, and had the function of “monooxygenase activity”. Among them, CYP1A1 and CYP1A2 had enhanced metabolic rates when induced by polycyclic aromatic hydrocarbons [24], while CYP3A22 may be the main contributor to xenobiotic metabolism in animal livers [25]. In the present study, these three CYPs were significantly up-regulated in SNT and LZT livers, suggesting that high-altitude environments are more prone to toxin production, which in turn induces toxicological responses in animal livers. Some studies have reported that CYP2E1 is most abundant in adult pig liver tissue and is able to metabolize many drugs and environmental toxins, playing a key role in the detoxification chain [26,27]. UGTs are a phase-II-specific family of enzymes that are capable of coupling and accelerating the metabolism of drugs and toxins. Nine UGTs from three Tibetan pig livers were screened as DEPs, namely, A0A286ZRB6, I3LJ68, F1RUQ4, F1SM17, I3LTQ3, F1SM28, A0A5G2QX61, I3LBU0 and A0A287B562. The expression of these UGTs in the livers of three groups of Tibetan pigs may be dominated by glucuronidation in the clearance of toxic compounds [28].

In addition, glutathione-S-transferase (GST) is an important detoxifying enzyme in phase II and is highly sensitive to stimulation by endogenous oxidative stress products and exogenous toxins [29]. A total of eight GSTs were found in the livers of three groups of Tibetan pigs, mainly including GSTA1, GSTA4, GSTK1, GSTM3, GSTO1, GSTP1 and MGST1. Compared with JZT, GSTA1, GSTA4 and GSTO1, expression levels were higher in SNT and LZT livers, which may be due to the fact that higher altitude requires greater expression of GSTs for enhanced detoxification in Tibetan pig livers, while GSTK1 and GSTM3 were significantly expressed in SNT livers, further suggesting that altitude can affect the sensitivity of the liver to exogenous toxins. Interestingly, GSTP1 is a key regulator of hepatocyte proliferation [30]. The expression level of GSTP1 (SNT/JZT, 0.541-fold; SNT/LZT, 0.699-fold) was significantly down-regulated in the livers of SNT under high-altitude environments, which may increase their liver damage and affect immune function. These results show that the detoxification function of Tibetan pig liver mainly comes from the function of an important mixed-function oxidase system in liver microsomes based on the change of protein expression level. However, the specific metabolic levels of these important enzymes need to be further analyzed and determined by metabonomics.

### 3.2. Lipid Metabolism Maintains Energy Homeostasis

**Figure 6:**
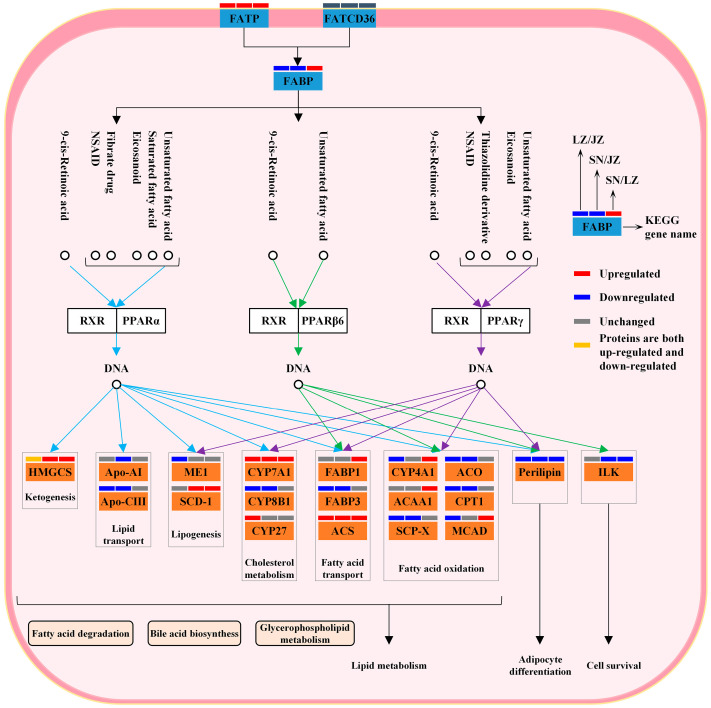
Expression of DEPs in “PPAR signal pathway”.

The liver is the central hub for lipid metabolism and is essential for maintaining the energy homeostasis of Tibetan pigs as they adapt to the highland environment. Previous studies have reported that peroxisome proliferator-activated receptor (PPAR), a steroid hormone receptor, is highly expressed in the liver and regulates intracellular lipid metabolism [31]. High-altitude animals are able to reduce lipid synthesis by inhibiting the PPAR signaling pathway, thus maintaining the energy homeostasis of the organism [32,33]. Quantitative proteomics showed that the “PPAR signaling pathway” was significantly enriched in three Tibetan pig liver KEGG pathways (Figure 4). This is consistent with the key proteins in this pathway also being significantly enriched in GO terms related to carboxylic acid metabolism and lipid metabolism (Table S3). Further, CD36 antigen and fatty acid transporter protein together regulate the expression of fatty acid binding protein (FABP), which in turn regulates the expression levels of target genes downstream of the PPAR signaling pathway (Figure 6). FABP3 (SNT/JZT, 0.752-fold; LZT/JZT, 0.644-fold) expression was down-regulated in SNT and LZT livers, indicating that the PPAR signaling pathway was inhibited in both types of Tibetan pig livers. Therefore, the levels of lipid metabolism downstream of the PPAR signaling pathway must be significantly different in Tibetan pigs from three different altitudes.

Lipid metabolism in the liver of three Tibetan pig species is mainly regulated by two isoforms, PPAR-α and PPAR-γ, in concert, including lipogenesis, fatty acid transport and fatty acid oxidation. Among the differential proteins associated with adipogenesis are malic enzyme (ME1) and stearoyl coenzyme A (SCD-1). ME1 (SNT/JZT, 0.831-fold; LZT/JZT, 0.738-fold), a class of enzymes that play an important role in the tricarboxylic acid cycle, had reduced expression activity in SNT and LZT livers; in contrast, pyruvate dehydrogenase A1 (SNT/JZT, 1.31-fold; LZT/JZT, 1.227-fold) and pyruvate dehydrogenase B (SNT/JZT, 1.402-fold; LZT/JZT,1.277-fold) were highly expressed in SNT and LZT livers. These results are consistent with earlier studies on broiler liver, where up-regulation of pyruvate dehydrogenase expression and downregulation of ME1 reduced the supply of acetyl coenzyme A, thereby inhibiting fatty acid synthesis [34]. SCD-1 (SNT/JZT, 5.968-fold; SNT/LZT, 6.114-fold) is the rate-limiting enzyme that catalyzes the synthesis of monounsaturated fatty acids, and this enzyme is significantly expressed in SNT livers, implying that SNTs would regulate lipid-stabilizing metabolism by increasing the expression level of SCD-1. Fatty acids are able to stimulate PPARα-induced peroxisome proliferation, fatty acid oxidation and ketone body production [35]. In this study, six DEPs were involved in the regulation of fat oxidation in the PPAR signaling pathway, among which carnitine palmitoyl transferase (CPT1), acyl coenzyme A oxidase (ACO) and sterol carrier protein (SCP2) played important roles in fatty acid oxidation. After catalytic activation by lipid acyl coenzyme A synthase, long-chain fatty acids are catalyzed by CPT1 and transported to the mitochondrial matrix for β-oxidation; ACO is both the initiating and rate-limiting enzyme for fatty acid β-oxidation, while SCP2 is a class of nonspecific lipid transport proteins that have been shown to bind phospholipids, fatty acids and fatty acyl coenzyme A with high affinity [36]. The expression activities of these three DEPs were significantly down-regulated in SNT and LZT livers, suggesting that SNT and LZT have higher levels of fatty acid oxidation than JZT and are able to provide more energy to cope with the alpine environment. In addition, hydroxymethylglutaryl-CoA synthase (HMGCS), a key enzyme for ketogenesis, was expressed more in the livers of SNT and LZT Tibetan pigs to increase ketone body synthesis and provide more fuel for liver peripheral tissues.

Among the PPAR signaling pathways, DEPs associated with adipocyte differentiation play an important role in Tibetan pigs coping with the hypoxic environment of the plateau. It has been reported that reactive oxygen species can promote lipid droplet formation in hepatocytes, especially PLIN2, through up-regulation of PLIN [37]. However, PLIN2 (SNT/JZT, 0.237-fold; LZT/JZT, 0.444-fold) was significantly down-regulated in SNT and LZT livers, suggesting that lipid droplet formation was inhibited in SNT and LZT livers compared with JZT livers at low altitude, which in turn reduced lipid accumulation in the liver. In addition, two other PLIN expressions were down-regulated in SNT livers: PLIN4 (SNT/LZT, 0.697-fold) and PLIN5 (SNT/JZT, 0.7-fold). Consistent with the role of PLIN2, low expression of PLIN4 was able to reduce lipid content in SNT liver, while reduced expression of PLIN5 may have enhanced fatty acid oxidative metabolism in SNT hepatocytes [38]. These results demonstrate that differences in lipid metabolism levels in Tibetan pig livers allow SNT and LZT to regulate body energy homeostasis better than JZT in response to the harsh environment of the plateau, which is closely related to the altitudinal environment in which they have lived for a long time. Of course, this view has only been inferred from the protein level and has certain limitations. The level of lipid metabolism will be further verified by other methods.

### 3.3. Immune Defense and Immune Regulation

The liver is not only a major metabolic organ, but also an important mediator of immune function, playing an important role in the defense of Tibetan pigs against viral and bacterial infections in the highland environment. It has been found that the extreme environment of high altitude affects the function of the immune system and makes it more susceptible to viral infections and organismal damage [39,40]. SNT and LZT live at a higher altitude, which greatly increases their degree of liver damage. However, the ability to activate immune-related proteins under hypoxia induction, which in turn affects the differential expression of three proteins that exert immune functions in the liver of Tibetan pigs, constitutes a specific immune defense system to maintain the immune capacity of the organism.

**Figure 7:**
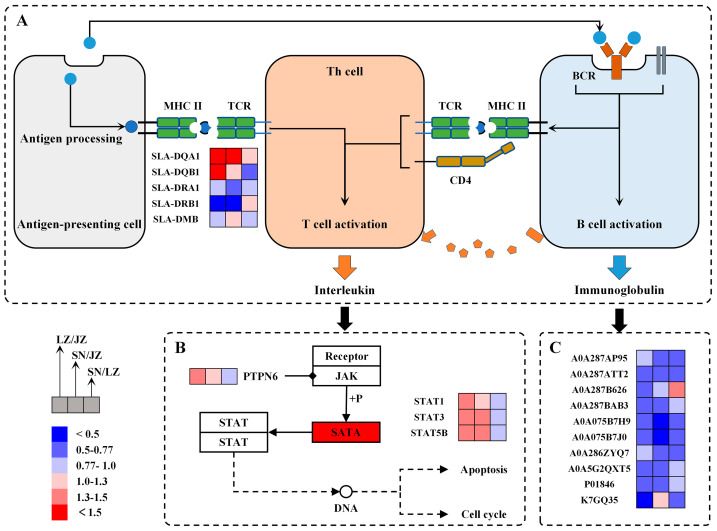
DEPs in immune-regulation-related pathways. (**A**) Antigen recognition and MHC II expression; (**B**) JAK-STAT signaling pathway; (**C**) immunoglobulin expression profile.

The major histocompatibility complex (MHC) is a group of genes that are closely associated with the immune response. During bacterial and viral infection of tissues, the MHC recognizes antigens and processes them for presentation to T cells, thereby activating an immune cascade response. Two main types of MHC molecules were identified in this study: MHC I and MHC II. Since MHC I is an endogenous antigen-presenting molecule that mainly recognizes and presents viruses to CD8+ T for paralysis, this implies that the ability of the SN Tibetan pig liver to defend against viral infection is enhanced mainly by up-regulating the expression of MHC I (SNT/LZT, 1.42-fold; SNT/JZT, 1.39-fold). In addition, MHC II is an exogenous antigen-presenting molecule, and in this study, five MHC II-like proteins were identified, four of which were screened as DEPs (Figure 7A). Two SLA-DQ proteins (F6PX38 and Q7YQ94) were expressed in high abundance in the livers of SNT and LZT, while two SLA-DR proteins (A0A286ZUJ9 and Q85ZW4) were less abundant in the livers of SNT and LZT. Previous studies reported that SLA-DQB1 is able to participate in the interaction with T cells in the immune response and may be a candidate gene related to disease resistance in pigs [41], while significant up-regulation of SLA-DQA in piglet organs was able to reduce transmembrane signaling of lipopolysaccharide from *E. coli* F18 and enhance humoral and cellular immunity [42]. In addition, SLA-DR is a key MHC II molecule involved in antigen presentation and initiation of CD4+ T cell activation, and downregulation of such molecules may lead to delayed host antibody responses [43,44]. Although it has not yet been determined whether these changes in MHC-related protein expression levels will be affected by changes in the number of immune cells, based on current proteomic analysis and research, the livers of Tibetan pigs at high and low altitudes show significant differences in the process of antigen recognition and presentation, which may affect the downstream signal and immune effect differently and requires further discussion.

The JAK-STAT signaling pathway can deliver signals to the nucleus in a minimum of steps, resulting in a rapid cellular immune response. T cells activated by antigen stimulation are able to express various cytokines, such as interleukins, interferons and growth hormones, which further activate the JAK-STAT signaling pathway. Specifically, cytokines can bind to the corresponding receptors to stimulate JAK, followed by STAT dimerization in response to JAK phosphorylation, which then crosses the nuclear membrane to regulate the expression of immune-related genes in the nucleus. Several studies have pointed out that after activation of the JAK-STAT pathway, up-regulated STAT1 inhibits macrophage proliferation during porcine reproductive and respiratory syndrome virus infection [45], activation of STAT3 benefits respiratory function and reduces hepatocyte injury in pigs [46] and STAT5 has a regulatory role in human complement regulatory protein CD46 on T cell proliferation and immunity in response to porcine aortic endothelial cells [47]. Notably, three STAT proteins were enriched in the KEGG pathway of the “JAK-STAT signaling pathway”, and the results showed that they were significantly expressed in the livers of SN and LZ Tibetan pigs compared to JZT, with no significant difference in SNT/LZT (Figure 7B). These different STATs are able to transmit signals from multiple cytokines to the nucleus, triggering different immune effects, especially in antiviral immunity. In addition, three protein tyrosine phosphatases (A0A286ZS66, A0A5G2QYP2 and A0A286ZLL2) were highly expressed in the livers of high-altitude Tibetan pigs, inhibiting JAK kinase activity and dephosphorylating STAT, resulting in a precise regulation of the immune response mediated by cytokines.

Antibodies are capable of removing antigens from the body and are primarily produced and secreted by activated B cells. It has been pointed out that immunoglobulins (Ig) are the most important immune molecules that exert immune functions in humoral immune responses. On the one hand, antigens bind directly to B cell receptors, thus stimulating B cells to secrete IgM and small amounts of IgG; on the other hand, cytokines secreted by activated T cells are able to act on B cells to produce other immunoglobulins. In this study, 33 Igs were identified, of which 10 Igs were differentially expressed (Figure 7C). In detail, an IgM (A0A287B626, 1.33) was significantly expressed in the livers of SN Tibetan pigs (*p* < 0.5), which may be a response of SN Tibetan livers to the virus in early immune defense. This is because IgM has a strong ability to activate complement while being produced or secreted early in the immune response. Interestingly, the expression levels of the other nine Igs decreased with altitude, and down-regulation occurred in both SNT and LZT livers. From the level of protein expression, this may be due to the high-altitude hypoxic environment suppressing immunity in mammals [39], or it may be due to the precise control of T cells over B cells, preventing the production of large amounts of antibodies and thus reducing the occurrence of autoimmune diseases.

## 4. Materials and Methods

### 4.1. Sample Collection

Six Tibetan pigs (300 days old) each from the Shannan, Linzhi and Jiuzhaigou regions of China were selected, respectively. The Tibetan pigs were euthanized by electric shock and slaughtered at a local slaughterhouse. Liver tissues for proteomics analysis were collected, frozen in liquid nitrogen and stored in liquid nitrogen tanks at −80 °C until analysis.

### 4.2. Protein Extraction and Digestion

Total protein extraction from Tibetan pig liver. A total of 5 g of Tibetan pig liver was and ground into powder, then 20 mL of lysis buffer (8 mol/L urea, 1% protease inhibitor mixture, 10 mmol/L dithiothreitol) was added. After thorough mixing, these sample solutions were sonicated (150 W, 30 s) three times on ice using a high-intensity ultrasonic processor (JY92-II, Ningbo Scientz Biotechnology Co., Ltd., Ningbo, China). After centrifugation at 4 °C for 10 min (12,000× *g*), the insoluble material was removed and the supernatant was collected. Protein concentrations were determined by a BCA kit (P0010, Beyotime Institute of Biotechnology, Shanghai, China).

Trypsin digestion of Tibetan pig liver proteins. An equal amount of total protein of the sample was taken, and the volume was adjusted consistently with lysis buffer after adding the appropriate amount of standard protein. The final concentration of 20% trichloroacetic acid was added and mixed well to precipitate at 4 °C for 2 h. Then, the mixture was centrifuged at 4500× *g* for 5 min, the supernatant was discarded, and the precipitate was washed with pre-chilled acetone 2–3 times. After the precipitate was dried, triethylammonium bicarbonate (TEAB) at a final concentration of 200 mM was added, followed by sonication to break up the precipitate, and trypsin was added at a ratio of 1:50 protease to protein, and digested overnight. Dithiothreitol was added to make its final concentration 5 mM, reduced at 56 °C for 30 min; next, iodoacetamide was added to make its final concentration 11 mM, incubated at room temperature and out of light for 15 min [15,48].

### 4.3. TMT Labelling and Fractionation

The enzymatically digested peptides were desalted and vacuum-freeze-dried by a Strata X C18 SPE column (Phenomenex). The thawed labeling reagent was dissolved in acetonitrile solution and mixed thoroughly with peptides that had been previously dissolved in 0.5 mol/L TEAB, incubated for 2 h at room temperature, then desalted and vacuum-freeze-dried. Peptides from SNT livers were labeled 126, peptides from LZT livers were labeled 128 and peptides from JZT livers were labeled 130. In addition, glutathione sulfotransferase and maltose binding protein from prokaryotes were added as standard proteins to assess the accuracy and reproducibility of the quantitative method. Quantitative information on peptides was obtained based on the abundance of labeled reporter ions.

The fractionation of labeled peptides was conducted using a high-pH reversed-phase liquid chromatographic separation scheme using an Agilent 300Extend C18 column (5 μm particle size, 4.6 mm inner diameter, 250 mm length) with a Waters ACQUITY UPLC (Milford, MA, USA). In brief, the peptide fraction was fractionated into 60 fractions in a gradient of 8% to 32% acetonitrile (pH 9.0) over 60 min, and subsequently randomly combined into 9 fractions, which were obtained by vacuum freeze-drying [49,50].

### 4.4. LC-MS/MS

Peptides were analyzed by nLC-NSI-MS/MS using a Q Exactive™ plus mass spectrometer with an EASY-nLC 1000 UPLC system. The labeled peptides were dissolved in mobile phase A (0.1% formic acid and 2% acetonitrile solution) and loaded onto a Reprosil-Pur C18 reversed-phase analytical column (1.9 μm particle size, 75 μm inner diameter, 15 cm length). The gradient of mobile phase B (0.1% formic acid and 90% acetonitrile solution) increased from 6% to 22% over 40 min, from 22% to 32% over 14 min, from 32% to 80% over 10 min, and then remained at 80% for the last 4 mi, with the flow rate maintained at 500 nL/min. The peptide segment was separated by an ultra-high performance liquid phase system, injected into an NSI ion source (voltage = 2.1 V) for ionization and then analyzed by full-scan mass spectrometry in the mass range of 350–1600 m/z with a resolution of 120,000, and for MS/MS analysis, with a resolution of 3000. Data were collected by a data-dependent scanning program, and the top 20 highest peptide parent ions were sequentially fragmented by a fragmentation energy of 28%. The automatic gain control was set to 1 × 10^5^, the signal threshold was set to 8.3 × 10^4^ ions/s, the maximum injection time was set to 60 ms and the dynamic exclusion time of the tandem mass scan was set to 30 s [48,51].

MS/MS data were retrieved by MaxQuant (v1.6.15.0) and compared with the database (Blast_Sus_scrofa_9823_PR_20201216.fasta; 49792 sequences). The mass error tolerance of the precursor ion was set to 20 ppm in the first search and to 4.5 ppm in the main search. Mass error tolerance for the secondary fragment ions was 20 ppm. Cysteine alkylation was designated as a fixed modification, and oxidation of methionine and acetylation and deamidation of the N terminus of the protein were designated as variable modifications. The quantification method was set to TMT-6plex, FDR was set to 1% for both protein and PSM identification.

### 4.5. Bioinformatics Analysis

The subcellular localization of identified proteins was predicted using WOLF PSORT (http://wolfpsort.seq.cbrc.jp/ (accessed on 16 January 2021)) with default settings.

Gene Ontology (GO) annotations of the identified proteins were obtained from the UniProt-Gene Ontology Annotation database (http://www.ebi.ac.uk/GOA (accessed on 16 January 2021)). The identified protein IDs were first converted to UniProt IDs and then mapped to GO IDs. Identified proteins in the UniProt-GOA database that were not matched with annotations were processed using InterProScan software for GO annotation based on protein sequence-matching methods. For GO enrichment analysis, a two-tailed Fisher exact test was used to test the enrichment of the identified modified proteins for all proteins in the species database, and GO terms with a corrected *p*-value < 0.05 were considered significant.

The pathways involved in the identified proteins were annotated using the Kyoto Encyclopedia of Genes and Genomes (KEGG) database by annotating the KEGG database descriptions of proteins using the online KAAS service tool, and mapping the enriched KEGG pathways based on the annotation information from the online KEGG mapping tool.

## 5. Conclusions

In this study, protein profiles were compared between SNT, LZT and JZT livers using TMT quantitative proteomics technology. A total of 1095 proteins were screened as DEPs. Bioinformatics analysis showed that the up-regulated DEPs in SNT and LZT livers were mainly involved in promoting liver detoxification through steroid hormone biosynthesis, maintaining body energy homeostasis by lipid metabolism, immune response and immune regulation, while the down-regulated DEPs were mainly involved in lipid metabolism and immune regulation compared with JZT livers. Endogenous harmful substances and exogenous toxins produced in a high-altitude environment can be effectively removed by the Tibetan pig liver itself, regulating important enzymes of oxidative metabolism. The PPAR signaling pathway was inhibited in SNT and JZT livers, thus reducing lipid synthesis and increasing fatty acid oxidation levels to provide energy to enhance organismal homeostasis in alpine low-oxygen environments in SNT and LZT livers. High expression in SNT and LZT liver proteins promote immune regulation, and ten differentially expressed down-regulated immunoglobulins suggest that JZT are more susceptible to autoimmune diseases. Although this research has certain limitations, it provides important information for Tibetan pig liver research at the protein expression level and helps to elucidate the molecular mechanisms of how the livers of Tibetan pigs respond to ecological environments at different elevations.

## Data Availability

The mass spectrometry proteomics data have been deposited to the ProteomeXchange Consortium via the PRIDE partner repository with the dataset identifier PXD039758.

## Supplementary Materials

The following supporting information can be downloaded at: https://www.mdpi.com/article/10.3390/molecules28041694/s1, Figure S1: The RSD boxplot (A) and Pearson’s Correlation Coefficient heat map (B); Figure S2: Subcellular localization (A) and COG/KOG annotation (B) of DEPs in LZT/JZT, SNT/JZT and SNT/LZT; Table S1: Detailed information of quantitative proteomic analysis; Table S2: The TMT labeling efficiency values; Table S3: RSD value of the sample; Table S4: Annotation information of differentially expressed proteins between SNT, LZT and JZT; Table S5: GO enrichment information of differentially expressed proteins in LZT/JZT, SNT/JZT and SNT/LZT; Table S6: KEGG enrichment information of differentially expressed proteins in LZT/JZT, SNT/JZT and SNT/LZT.
