# Supplementary figures and images for: Quantitative Proteomic Analysis of Tibetan Pig Livers at Different Altitudes
Source: Molecules. 2023 Feb 10;28(4):1694. doi: 10.3390/molecules28041694 (PMC9960092; doi:10.3390/molecules28041694)

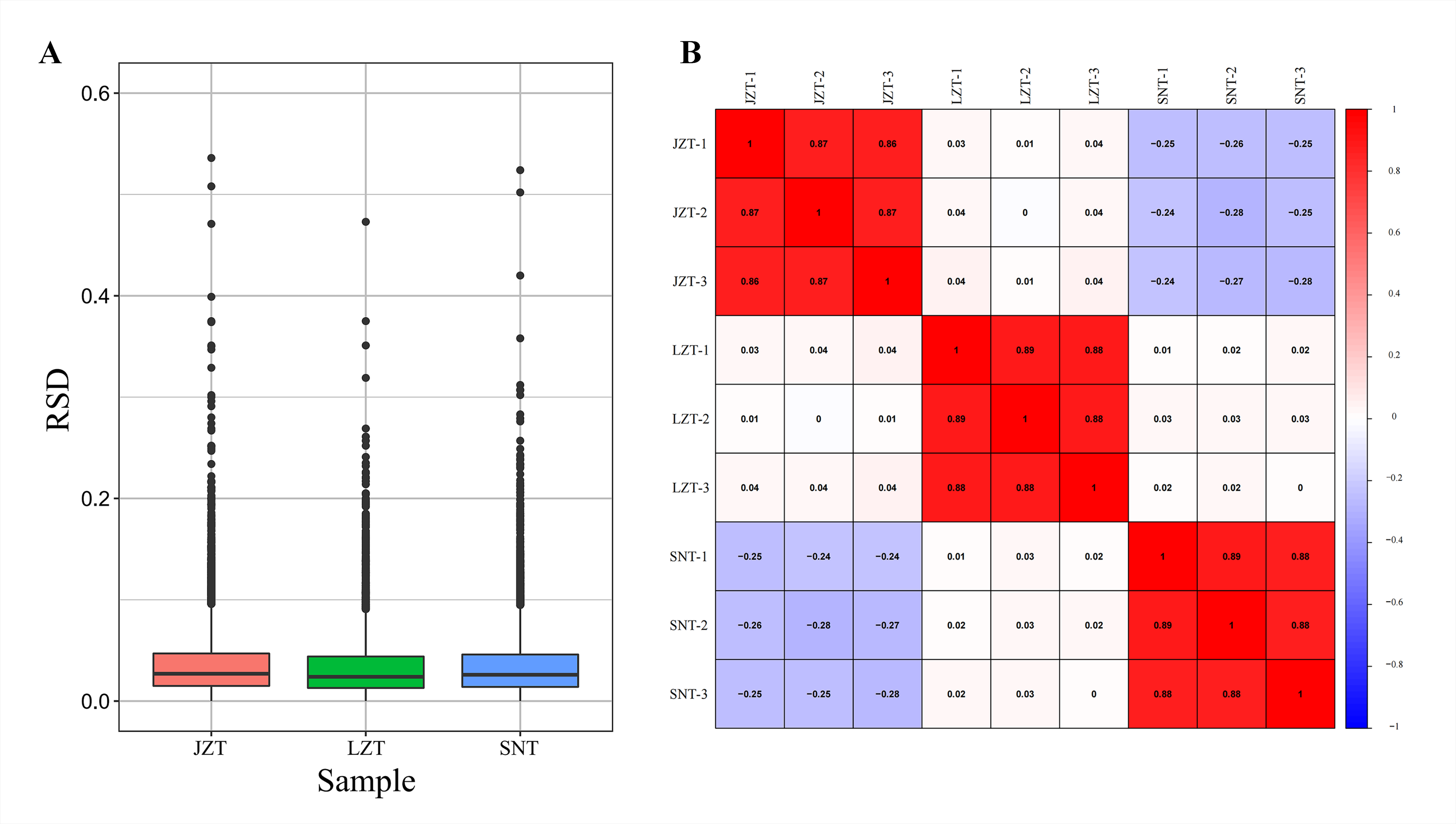

Supplement: Supplementary file 1 [file molecules-28-01694-s001.zip › Figure S1.tif]

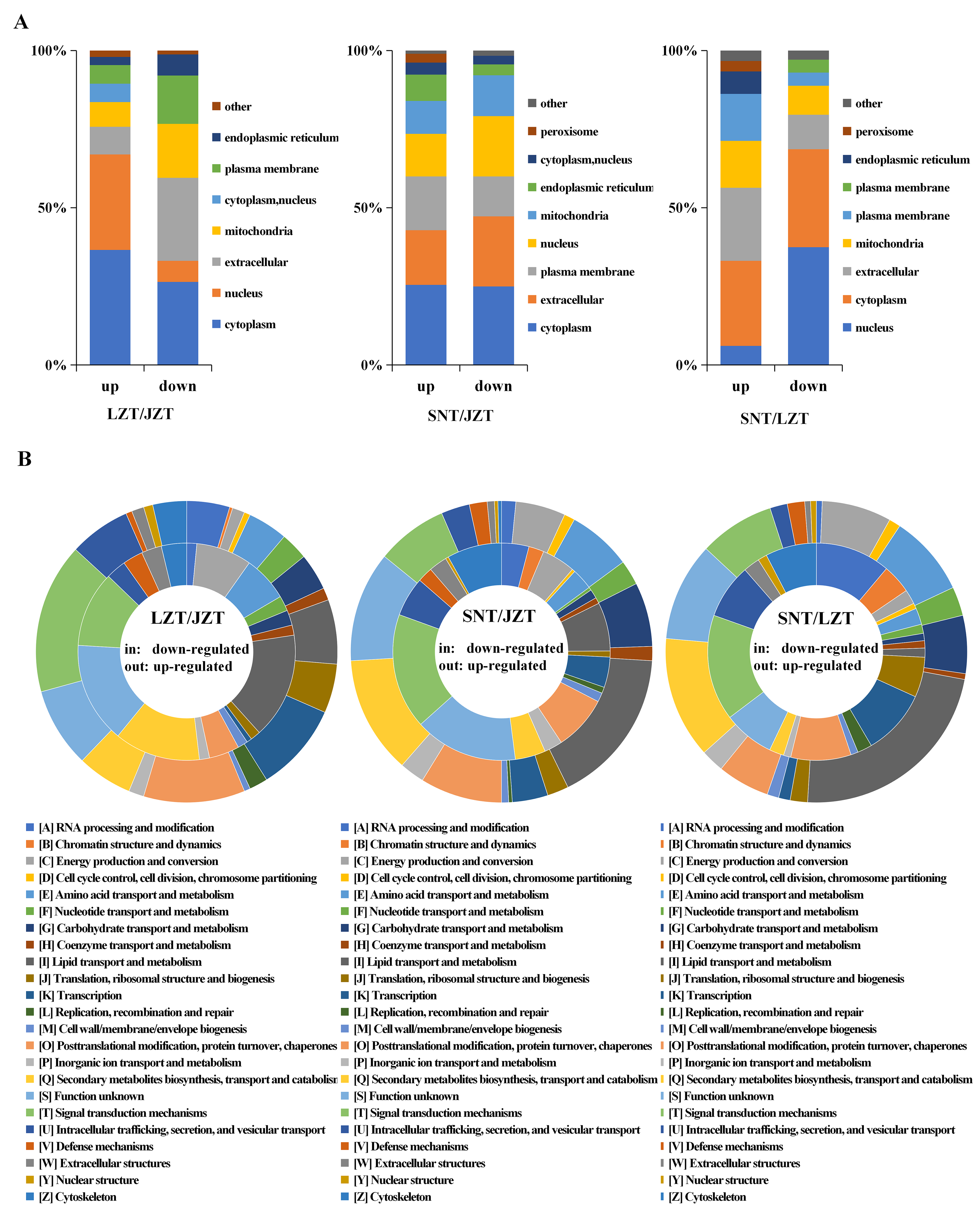

Supplement: Supplementary file 1 [file molecules-28-01694-s001.zip › Figure S2.tif]
